# Re-evaluating model assumptions suggests that Australian birds are more tolerant of heat and aridity than predicted: a response to Conradie *et al*. (2020)

**DOI:** 10.1093/conphys/coac010

**Published:** 2022-03-10

**Authors:** Hector Pacheco-Fuentes, Christine E Cooper, Philip C Withers, Simon C Griffith

**Affiliations:** School of Natural Sciences, Macquarie University, Sydney, New South Wales 2109, Australia; School of Molecular and Life Sciences, Curtin University, Perth, Western Australia 6102 Australia; School of Biological Sciences, University of Western Australia, Crawley, Western Australia 6009, Australia; School of Natural Sciences, Macquarie University, Sydney, New South Wales 2109, Australia

## Abstract

Conradie *et al.* (2020) recently modelled the vulnerability of Australian arid birds to a changing climate. While the approach used by Conradie *et al.* (2020) is valuable, we argue that key assumptions in their study are poorly supported and the risks of a changing climate to arid zone avifauna are consequently overstated.

Conradie *et al.* (2020) recently evaluated the risk of hyperthermia and dehydration for 10 species of Australian desert bird to projected climates, concluding that small species like the zebra finch (*Taeniopygia guttata*) were likely to decline throughout their range as a consequence of escalating lethal dehydration risk and hyperthermia. Their modelling was based on birds’ physiological response to air temperature (T_a_) under experimental conditions in a laboratory relative to existing and projected climate throughout the Australian arid zone (Conradie *et al.,* 2020). They predicted that smaller (10–42 g) bird species, particularly those with a westerly distribution, would be severely impacted by the end of the century, with the zebra finch identified as a species especially at risk, in the north-west of its range. While we agree that it is important to model the likely effects of climate change on the persistence of birds, and that a more extreme climate will likely impact on their abundance and distribution, we believe that the predicted outcomes underestimate the capacity of birds to withstand heat and aridity and consequently may overestimate the likely physiological effects of environmental change. Indeed, we have observed several species, including the zebra finch, withstanding predicted lethal limits in the field (e.g. zebra finches persisting when T_a_ > 46°C and with a maximum air temperature of 47.5°C; at Fowlers Gap, New South Wales, on the following dates: 16, 17 and 24 and 25 January 2019 and 29 November 2020; data from the Bureau of Meteorology Automated Weather Station #46128), despite the predicted dehydration and thermal limits of 41.5 and 46°C, respectively, (Conradie *et al.,* 2020). Consequently, we will address the assumptions of Conradie *et al.* (2020) that most likely contribute to this discrepancy. We hope that the perspective we provide concerning the assumptions of predictive models improves the accuracy of future models and encourages a more comprehensive integration of laboratory and field observations of physiology and behaviour. This, combined with steps to ground-truth predictive models, will contribute to a better understanding of ecological physiology and its application to conservation and management in the face of climate change.

## Assumption: water is unavailable to birds throughout much of Australia’s arid zone

The Australian arid zone is largely defined by low rainfall (Morton *et al.,* 2011), along with high rates of evaporation and high variability of rainfall and T_a_ (Bradshaw, 1986). Conradie *et al.* (2020) suggested that unavailability of water limits drinking for birds during hot periods and declining rainfall would further reduce access to drinking water under future climate scenarios. However, standing water is abundant throughout much of Australia’s arid zone, with the presence of natural water points currently reflecting their past distribution (Bird *et al.*, 2016). Even if these water sources become more saline during dry periods, desert birds still drink (Skadhauge and Bradshaw, 1974). Rainfall is predicted to decline in some parts of Australia as a consequence of human-mediated climate change, as recognized by Conradie *et al.* (2020), but the study they cited (Delworth and Zeng, 2014) predicts increasing rainfall in the north and north-west of the continent and the IPCC (2018) predicts only minor (0–5%) changes to mean rainfall for this region, but with increased variability. Therefore, there is unlikely to be less natural water available to birds in general in the region that Conradie *et al.* (2020) predict zebra finches to be most at risk from climate change. Indeed, higher rainfall has been observed in that region since 1981 (Davies *et al.*, 2010; Delworth and Zeng, 2014) with a shift to increased summer rain (Hughes, 2003), which means water is more likely available when birds’ thermoregulatory demand for water is highest.

While the availability of naturally occurring water is largely driven by climatic variation, the Australian arid zone has been enriched with a high density of groundwater-fed artificial water points to support livestock (Fensham and Fairfax, 2008; James *et al.*, 1999). Rangeland pastoralism is the predominant land use over 70% of Australia’s arid zone (James *et al.,* 1999; Pickup, 1998) and in these areas there are few locations more than 5–7 km from water (Davies, 1972; Davies *et al.,* 2010; Fensham and Fairfax, 2008; James *et al.,* 1999; Pickup, 1998); water-dependant birds typically remain within 12 km of a water source but can travel up to 20 km to water (Harrington, 2002). These artificial water points are consequently a reliable water supply for fauna (Florance *et al.,* 2011; Letnic *et al.,* 2015; Letnic *et al.*, 2014) and water is widely available *ad libitum* to most mobile animals throughout at least 70% of the arid zone (James *et al.,* 1999). Of note, the density of natural and artificial water points characterized in Fig. 2 (James *et al.,* 1999), Figure S1 of Bird *et al.* (2016) and Figure 1a and b of Davies *et al.* (2010) is particularly high in the areas projected by Conradie *et al.* (2020) to be most challenging for zebra finches in the future, i.e. in the areas that will be hottest in the future there is also predicted to be a relative abundance of water, with the exception of the Simpson and Great Sandy deserts. Closure of watering points to reduce grazing pressure on pastoral lands converted to conservation reserves could potentially affect birds, but this issue has been recognized and watering points may be retained to maintain biodiversity (Croft *et al.*, 2007), with fencing as an alternative approach to manage grazing by introduced herbivores (Graz *et al.*, 2012).

**Figure 1:**
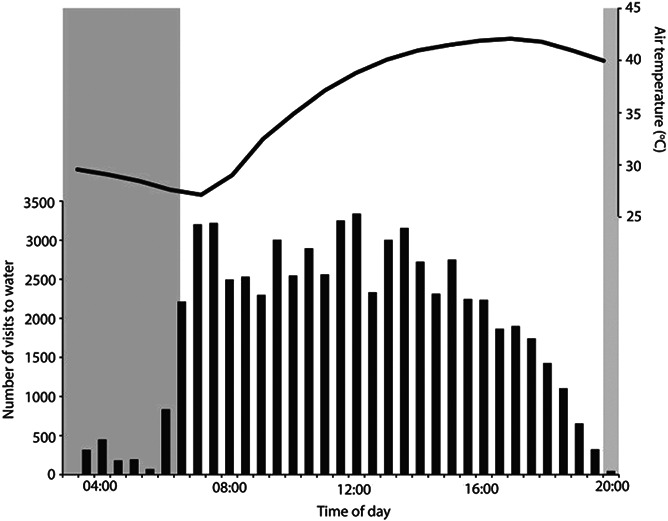
Frequency of visits to water troughs at 30-min intervals throughout the day by a population of approximately 350 zebra finches (*Taeniopygia guttata*) for 17 days of maximum air temperature of >40°C between 1 December and 17 February 2018, together with mean hourly air temperature (black line) from Australian Bureau of Meteorology for Fowlers Gap (station number 046128). Grey panels indicate the period from sunset to sunrise; solar radiation increases from sunrise to peak at approximately midday, declining to 0 at sunset (Kondragunta *et al.,* 1993). Figure re-drawn from the data of Cooper *et al.* (2019).

## Assumption: birds do not drink to replace water lost during periods of extreme heat

It is a common fallacy that high rates of evaporative water loss are unstainable. If evaporated water is replaced by drinking, then evaporative heat loss can be sustained, although it is more challenging for small species (Mitchell *et al.,* 2018). Conradie *et al.*’s (2020) calculations of dehydration tolerance assumed that birds do not replace water lost at high T_a_. Even their most conservative model, which assumed that the birds started with a crop full of water, did not allow for birds drinking throughout the day, due to the assumption that birds do not have access to water and if they do, they avoid activity and exposure to solar radiation at high T_a_. However, water-dependant Australian species such as many granivores (including the zebra finch) and nectarivores are typically nomadic and only persist in close proximity to water (Davies, 1984; Harrington, 2002; Burbidge and Fuller, 2007; Jordan *et al.*, 2017). Indeed, water-dependant birds such as the zebra finch are reliable indicators of proximity to water in Australia’s arid regions (Bayly, 1999). We established that birds inhabiting the majority of Australia’s arid zone have ready access to drinking water. We now demonstrate that they use this water throughout the day.

It has long been appreciated that many of quintessential Australian desert birds, including the iconic budgerigar (*Melopsittacus undulatus*) and zebra finch, are water dependant on days when T_a_ ≥ 25°C (Fisher *et al.*, 1972). Zebra finches, along with other arid-habitat birds including painted finches (*Emblema pictum*), diamond doves (*Geopelia cuneate*) and spinifex pigeons (*Geophas plumifera*) drink throughout the day on hot days (Davies, 1982). Cooper *et al.* (2019) established that zebra finches visit water to drink throughout the day during heatwaves on days with a maximum T_a_ of up to at least 44.5°C, and found no evidence of zebra finches having to withstand any considerable time without access to water (Fig. 1). Solar radiation in arid environments increases in intensity throughout the day, peaking around midday and then declining to 0 at sunset (and there is a lag in the diel T_a_ cycle compared to the radiation cycle due to thermal inertia and radiative cooling; Kondragunta *et al.*, 1993). For a population of approximately 350 zebra finches of which a proportion were implanted with an identification Passive Integrated Transponder (PIT tag), there was a high frequency of visits to water between sunrise and sunset, with especially high drinking rates prior to 15:00 (Cooper *et al.,* 2019; Fig. 1), during the period when solar radiation is most intense. Indeed, finches visited water more frequently during the middle of the day on hot days than they did on cooler days, to sustain the higher water intake required to counteract a lower metabolic water production during hot conditions (Cooper *et al.,* 2019). Regular access to drinking water allows birds to maintain sufficient evaporative heat loss to defend a lower body temperature at high T_a_ compared to dehydrated birds (e.g. Arad *et al.*, 1987; Crawford Jr and Schmidt-Nielsen, 1967; Itsaki-Glucklich and Arad, 1992), reducing the likelihood of fatal hyperthermia.

## Assumption: operative temperature experienced by a bird is equivalent to air temperature

Many authors (e.g. Gerson *et al.*, 2014; Hetem *et al.*, 2016; Williams and Tieleman, 2005; Withers *et al.*, 2016; Wolf and Walsberg, 1996) state that evaporation is the only avenue of heat loss for animals when T_a_ > T_b_. They assume that T_a_ is equivalent to operative temperature (or more correctly environmental temperature; T_e_), as do Conradie *et al.* (2020)—‘the operative temperature experienced by the bird is equivalent to air temperature’. However, T_e_ incorporates evaporative, radiative, conductive and convective avenues of heat exchange (Bakken, 1976; Bakken *et al.*, 1985) and, consequently, T_e_ not T_a_ determines heat exchange (Mitchell *et al.,* 2018; Wolf, 2000). For an animal in a temperature-controlled cabinet or room, the assumption that T_a_ approximates T_e_ is reasonable; the thermal limits that Conradie *et al.* (2020) identified are realistic for birds under these laboratory conditions, as there is no object in the animal’s environment with a temperature less than T_a_. The problem arises when extrapolating laboratory findings to wild, free-living individuals where the assumption that T_a_ = T_e_ is unrealistic (Maloney and Dawson, 1994) due to the complexity of the animal’s thermal environment. This can substantially distort heat tolerance calculations (Mitchell *et al.,* 2018).

Heat loss via radiation to objects cooler than T_a_ and selection of cool microclimates where T_e_ < T_a_ are two examples of how an animal may potentially lose, not gain, heat when T_a_ > T_b_ without augmenting their own evaporative heat loss. The significance of radiative heat loss to the night sky, which is considerably lower than T_a_ and often approaches the mean troposphere temperature of −23**°**C (Sun *et al.*, 2017), has long been appreciated, e.g. Schmidt-Nielsen’s camels (Schmidt-Nielsen *et al.*, 1956). However, not so widely appreciated by biologists is that daytime sky temperatures can also be considerably lower than T_a_. Clear, dry desert day-time skies are typically >20°C lower than T_a_ and can be a significant heat sink (Adelard *et al.,* 1998; Evangelisti *et al.*, 2019). Thus, net radiative heat exchange with the daytime sky can be a potential source of heat loss for animals in their natural environment even when T_a_ > T_b_.

**Figure 2:**
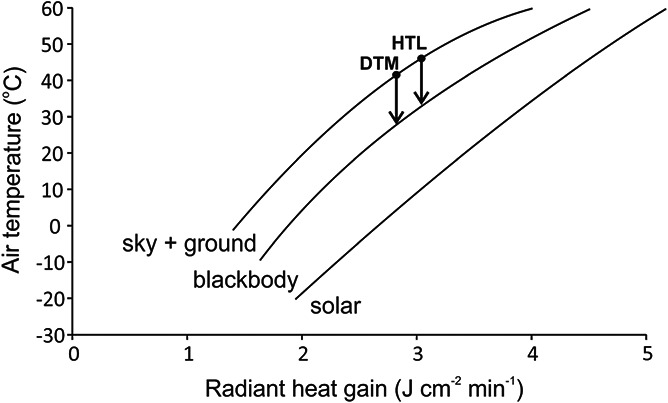
The relationship between air temperature and radiative heat gain modelled for the zebra finch (*Taeniopygia guttata*; modified and extrapolated from Porter and Gates, 1969) for three environmental conditions; sky + ground (i.e. natural shade environment), blackbody (i.e. metabolic chamber) and solar (i.e. with direct solar radiation). The dehydration thermal limit (DTM) of 41.5°C and the heat tolerance limit (HTL) of 46°C (Conradie *et al.,* 2020) measured at a sky + ground air corresponds to equivalent radiative heat gains at temperatures in a metabolic chamber of 30–33°C.

We can use a simple heat balance model for zebra finches (Porter and Gates, 1969) to estimate the cooling effect of daytime sky temperatures (Fig. 2). If net radiative exchange is constant, then a bird in a metabolic chamber (surrounded by a black bulb = T_a_) is effectively at a higher T_a_ than the bird in its natural environment sheltered from solar radiation but experiencing heat loss to the sky (surrounded by a combination of ground black bulb = T_a_ and daytime sky grey bulb at <T_a_). At T_a_ = 41.5°C (calculated dehydrational maximum for a zebra finch; Conradie *et al.,* 2020), the heat loss of a bird in natural shade facilitated by radiation to the daytime sky, is equivalent to T_a_ of ~30°C for a bird in a metabolic chamber. At the calculated lethal heat T_a_ of 46°C, the equivalent natural T_a_ is only ~33°C.

Birds can also exploit other aspects of their environment to achieve heat loss without augmenting their own evaporative heat loss at T_a_ > T_b_. Conductive loss is possible if the animal can contact a surface cooler than T_b_, and radiative loss will occur to objects with a surface temperature < T_b_. Briscoe *et al.* (2014) suggested that cool trees can be a heat sink for many animals including birds, and Wolf *et al.* (1996) calculated that small desert birds reduced evaporative water loss by 50–66% by sheltering in hollows or against the trunks of trees that were ~3°C < T_a_. Evaporo-transpiration can cool the immediate environment within tree canopies (Rahman *et al.*, 2017) and we have observed zebra finches sheltering in shade under dripping stock troughs during a recent heatwave; T_a_ > 46.5°C but T_e_ experienced by the finches was likely lower due to evaporative cooling of their immediate environment (Griffith, pers. obs.). Sheltering in these favourable microclimates presumably imposes costs associated with foraging, reproduction and predation, but an observation of birds persisting during periods of extreme T_a_ is evidence they can balance these costs. Our data for body mass and blood parameters of wild, free-living zebra finches indicates that withstanding T_a_ of up to 45.2°C does not impose a significant physiological cost (Cooper *et al.,* 2019, 2020b).

## Assumption: physiological traits that determine thermal tolerance are fixed

Physiological change is an important but often overlooked component of animals’ response to environmental change (Mitchell *et al.,* 2018; Wojciechowski *et al.*, 2021). Animals can respond to changing environmental conditions by microevolution and by developmental or acclimatory plasticity. Failing to consider these responses in mechanistic climatic models produces inaccurate predictions (Clusella-Trullas *et al.*, 2021; Fuller *et al.,* 2010). There are a plethora of studies demonstrating adaptive and plastic physiological change by birds in response to environmental change, including zebra finches. In addition to an array of genetic, reproductive, morphological and behavioural effects of captivity (e.g. Forstmeier *et al.*, 2007; Gilby *et al.*, 2013; Mainwaring *et al.*, 2010; Tschirren *et al.*, 2009). Skadhauge and Bradshaw (1974) and Cooper *et al*. (2020a) reported that captive conditions impact on the metabolic and hygric physiology of zebra finches, demonstrating physiological consequences of differing environmental conditions. Developmental plasticity in response to exposure to, or signals of, high T_a_ impacts body mass and growth (Andrew *et al.*, 2017) of zebra finches. There is also considerable evidence of phenotypic plasticity; zebra finches respond to chronic and acute acclimation and acclimatization to varied T_a_ and water availability by adjusting reproductive, cellular and thermal physiology, including metabolic heat production, evaporative heat loss and/or T_b_ (e.g. Salvante *et al.*, 2007; Niedojadlo *et al.*, 2018; Cooper *et al.,* 2019, 2020a, 2020b; Szafrańska *et al.*, 2020; Wojciechowski *et al.,* 2021).

Cooper *et al.* (2019) demonstrated that the ability of zebra finches to predict periods of extreme T_a_ assisted them to withstand these conditions by implementing appropriate behavioural and physiological responses. As climate change realizes projected future T_a_ increases, the likelihood that birds will have already experienced extreme conditions will be greater, improving resilience of zebra finches and facilitating favourable plastic responses (Cooper *et al*., 2020a). An inability to predict and respond favourably to unexpected extreme events is one factor hypothesized to contribute to heat-induced avian mass mortality (Cooper *et al.,* 2019). Attempts to model future responses of birds, including small passerines such as the zebra finch, to changing environmental conditions must consider their demonstrated capacity for micro-evolutionary or plastic adaption. In summary, we feel that the overall conclusions of Conradie *et al.* (2020) are overstated and that the degree to which Australia’s arid zone birds are vulnerable to the changing climate is currently unclear. Predicting the vulnerability of species to climate change is difficult, but a worthy objective and can be improved through a comprehensive consideration of physiological, ecological and evolutionary processes (Clusella-Trullas *et al.,* 2021).

## Funding

This work was supported by the National Agency for Research and Development of the Republic of Chile—International Macquarie Research Excellence Scholarship (BECAS Chile/International Doctorate Scholarships 2019-72200260 to H.P-F.) and the Australian Research Council’s Discovery Project funding (DP170103619 to S.C.G. and C.E.C.).
